# My Dissection Wound and What Came of It

**Published:** 1863-04

**Authors:** 


### MY DISSECTION WOUND AND WHAT CAME OF IT.

Dissection wounds and death are so commonly associated, that we would as reasonably expect an autographic obituary notice, as a similar sketch of a dissection wound. Notwithstanding this uniform fatality, I have had the pleasure of a very close interview with his grim majesty, and am still left to tell of it. If every one keeps in as "good spirits" as I did, the terrors of death must be very much exaggerated. In fact, I felt that while Buck & Rayner's S. O. P. lasted I should have no occasion to conjure them from the vasty deep, and were it not non-professional would be tempted not to give my treatment, lest the accident become *epidemic*.

Mr. Van ——— of this city, became entangled in the gearing of his mill, while in motion, was injured, and kindly referred to me for surgical treatment by Prof. Miller.

I saw him first on the third of February, 1863. Upon examination, found his right arm fractured at the union of the lower and middle thirds, the radius broken at the union of its middle and lower thirds, and the ulna at the junction of its upper and middle, the latter a compound fracture, the parts around being considerably bruised. Besides he had some trifling bruises on the lower right side of his chest. The patient was unusually stout and robust, of the sanguino-lymphatic temperament, always enjoying the best of health.

Assisted by Drs. Shumway & Lynn, I dressed the fractures with splints lightly applied, leaving an open space about the compound fracture, and gave the patient an anodyne.

Saw him the next day, found he had slept tolerably well during the night, and having taken the anodyne pretty freely was quite free of disturbance. We moved him to his uncle's, on a bed in one of our street cars, the distance being about a mile, which was made easily and with little or no discomfort.

I then removed the dressings, and as the lower fragment in the compound fractuie was very sharp, I clipped off its point with my bone forceps, in order to allow it to rest more firmly against the upper fragment and act as an extending splint, but it came nearer performing the same valuable service for me, for while doing so, under the guidance of the left forefinger, the forceps slipped off and cut it slightly, not more than two lines, but seemed to bruise it very much. It hurt me much, and I immediately put it in warm water and squeezed it, causing it to bleed freely.

I redressed the limb lightly as before, and thought but little of my autoplasty until near night, when it began to feel very tender, which gradually increased to a deep painful throb confined to the end of the finger. The pain continued through the night, so that I rested but little, keeping my finger in a basin of ice water most of the time, though I was slow to believe that it was more than the bruise of the forceps that was causing the trouble.

An examination in the morning dissipated any doubts I might have had of its character, and I was placed in front of that danger which always seemed a myth, from its far nearness. Eight years in the presence of a danger does much to dissipate the dread of it, and induces the carelessness likely to produce it. My finger was swollen, tense, red, throbbing, the pain extending to the elbow and shoulder, with two red lines of lymphatics passing from the back of the hand to the elbow, and a blush on the back of the hand at the point of beginning of the inflamed lymphatics. I felt a sense of languor similar to that preceding an attack of ague, with the attendant aching of my limbs and indisposition to exertion.

At 10 o'clock, I cauterized the finger thoroughly, and Dr. Lynn split entirely to the bone the length of the last phalanx through the wound. It bled probably a drachm or two, after which it felt somewhat relieved, and wrapping it in a linen roller, I attended to my business during the forenoon. Getting home at one o'clock, and my hand in a perfect rampage of pain, as I entered the house was taken with a violent chill, which shook me *entirely*, until I thought I should disjoint without the benefit of the clergy. This did not continue more than fifteen minutes, and was followed in about an hour by fever my pulse rising to about 120, which with alternate cold flashes continued the afternoon.

When I was taken with the chill I took a very free dose of brandy, and repeated the dose in half an hour. If any one is curious to know the amount I must refer them to my kind and frightened landlady, who dealt it with no sparing hand. As I came from my office at 3 o'clock, I ordered a quart bottle of Buck & Rayner's best, and began on it immediately, and continued it at *uncertain* short intervals during the afternoon. Shortly after 6 o'clock I broke into a profuse perspiration, which lasted but a few minutes. After this, my fever seemed to rise higher. ℞. Cont. Brandy, Pro re nata, and more too. At bed time, Blue Mass, grs. X. At midnight, I began to perspire freely, which continued through the night. Next morning found me free of fever, my finger comparatively comfortable; did not examine the wound.

℞. Cont. Brandy, slightly increasing the dose, from the encouraging effects thus far. Took a bottle of Citrate of Magnesia which operated freely.

No increase of fever during the day.

Night found my medicine exhausted as well as the fever, both going down together. In the evening my finger pained me some, which by 2 o'clock A. M. became so unbearable that I opened it to find the wound suppurating slightly, and erysipelas extending the length of the finger, swelled to the back of the hand.

Cauterized it with solid Nitrate of Silver, and applied bread and milk poultice to the wound, and it becoming easy, I slept well, except when I roused myself to sustain my flagging powers with a toast to the inventor of agreeable medicines.

Morning found me with another empty bottle, this time only Oj., and my finger seemingly free from further anxiety, the erysipelas ceasing to spread and pain almost having disappeared. This day, Saturday, I went to my office, though feeling very weak. After this there is nothing in the history of the case of any interest, if there is any before.

Erysipelas, followed by gangrene, set up in my patient's arm and he died the fifth day.

Aside from the *personal* interest I have had in the case, there seems to me an interesting inquiry arises, which I have no hope of settling, although that does not serve to keep it down. Why should I escape so many similar exposures to be overtaken this time? Leaving out the theological view, which I firmly believe, "Whatsoever comes to pass," &c., there is much mystery about the *matter* of infection. Many times, so far as extreme appearances could go for any thing, I must have been exposed to all the reputed causes of this uniformly fatal accident.

Often I have cut my fingers in dissecting, an accident occurring every day almost, and on almost all kinds of subjects, and never had the slightest symptom of any thing of the kind before. Generally, the caustic was applied, but if the ideas of some of our latest authorities go for much this is of uncertain avail, for they maintain that once the infectious material within reach of the absorbents no power can rescue the contamination. In vaccination, it is maintained, that when once applied, though scarified, cupped and washed with chlorinated water, the disease was not interfered with in the slightest. Glanders has been introduced into the system in the same way, though followed by excision of the part and the actual cautery. Very often this was not done, and every thing was left to its natural course with perfect impunity. This was not so alone with the cadaver, but also on living subjects.

While on the hospital boat from Pittsburg Landing, I recollect *knocking* the skin off my fingers in opening some boxes and afterwards using them probing erysipelatous wounds and handling cases of hospital gangrene, without having the slightest inconvenience from it.

Wherein does the predisposition exist? Is it in the poisoner, or poisoned. It seems to me in the latter, from the facts I have just stated. The condition of the system at the time it is exposed to the infection determines its reception or rejection; not its absolute acceptance or rejection, but its deleterious effect. In certain conditions of the blood and organs, any deleterious agents received within due limits may be eliminated, while in others, either from the blood proving a suitable and comfortable nidus, or from the organs whose business it is to throw [illegible]

off being imperfect in practice, it is retained, and all the consequences entailed. And here exists one of the great predispositions to disease, the condition of the blood and eliminating organs.

One case of confinement does well, or all cases do well in one season, and in another with no outward difference in the cases many are affected with fever and none do so well as formerly. In one season the system, with its usual energy, purifies itself and speedily regains its accustomed vigor, and in another, from the blood being contaminated or contaminatable, the resolvent actions are impeded, putrid or stagnant blood received into the circulation, is sufficient to light up the fires of fever. In one season or case a given injury readily rights itself with no serious symptoms, while in another gangrene or erysipelas complicates or destroys.

In scarcely one out of ten cases of Gonorrhœa treated, is the patient made acquainted with the danger of contact of the infectious matter with the conjunctiva, and he handles organs and eyes with indiscriminate ignorant freedom and yet how rare is purulent opthalmia?

I think that those blood diseases will be found to prevail during epidemics of erysipelas and childbed fever, and to this fact, and not contagion, are these epidemics to be attributed, that it is a coincidence and not a cause.

In one season the diseases of children, as scarlet fever, measles, &c., are comparatively light, scarcely sufficient to keep the patient indoors; while during the present winter very many died, and that too under the most skillful treatment. I have noticed the present year, as a collateral fact, that the cases I have vaccinated have been unusually successful in taking, their arms excessively sore—in many cases, though I always used the purest matter, an eruption, pustular in character, would break out on the arm—the surface around it remain livid and the arm not heal for four or six weeks. These facts evidence to my mind that there are times when, from the altered relations of the elements, some additional elements, or the changed eliminative power of the excretory organs, the blood receives injurious impressions with more facility.

A person may be exposed for years to the same exciting causes of disease with entire immunity, until the two poles meet with the necessary conditions for an explosion. I know I am stepping aside from the usual idea in relation to this subject, it always being accounted as depending on the character of the infecting matter rather than condition of the individual receiving it. Certain diseases, as Puerperal and Erysipelatous, being those regarded as most dangerous, the fact of their prevalence is, I believe, evidence of the epidemic influence causing depraved condition of the blood. Or were they not prevailing it would weigh nothing against the circumstances of an individual case being such as to cause the necessary depravation of the blood, as from exposure, bad food, or great mental and bodily fatigue.

The treatment suggested itself to me as that most successful in other forms of animal poisoning, and as so little was to be expected of those usually adopted there seemed but little risk in trying it, with the additional advantage of being a pleasant medicine and a great comfort to the suffering. I gave it a very free trial, having taken three pints of the best brandy in thirty-six hours, which for one not a politician may be considered fair drinking. The test of its efficacy in this disease is said to be its want of effect on the mind. The only circumstance from which I could judge was getting well, though I noticed occasionally a disposition to converse freely, but I took the precaution not to apply the test of locomotion. I feel much confidence from my experience in the remedy, and should use it again with great hopes under similar circumstances, the very best test of its power being to me that I am able to subscribe myself R**.

P. S.—Since writing the above, Dr. Chas Fishback, of Indianapolis, has lost his life by this accident. He was making a *post mortem* when he pricked his finger slightly with a needle, the disease set in, destroying his life. I have seen nothing but a newspaper notice of the case and, therefore, know nothing of its treatment or duration.

